# A 61% lighter cell culture dish to reduce plastic waste

**DOI:** 10.1371/journal.pone.0216251

**Published:** 2019-04-30

**Authors:** Pedro Réu, Gustav Svedberg, Lars Hässler, Björn Möller, Helene Andersson Svahn, Jesper Gantelius

**Affiliations:** 1 KTH Royal Institute of Technology, School of Engineering Sciences in Chemistry, Biotechnology and Health, Department of Protein Science, Stockholm, Sweden; 2 KTH Royal Institute of Technology, School of Industrial Engineering and Management, Department of Machine Design, Mechatronics, Stockholm, Sweden; Universiti Putra Malaysia, MALAYSIA

## Abstract

Cell culture is a ubiquitous and flexible research method. However, it heavily relies on plastic consumables generating millions of tonnes of plastic waste yearly. Plastic waste is a major and growing global concern. Here we describe a new cell culture dish that offers a culture area equivalent to three petri dishes but that is on average 61% lighter and occupies 67% less volume. Our dish is composed of a lid and three thin containers surrounded by a light outer shell. Cell culture can be performed in each of the containers sequentially. The outer shell provides the appropriate structure for the manipulation of the dish as a whole. The prototype was tested by sequentially growing cells in each of its containers. As a control, sequential cultures in groups of 3 petri dishes were performed. No statistical differences were found between the prototype and the control in terms of cell number, cell viability or cell distribution.

## Introduction

Plastic waste is a major and growing concern around the world. The oceans alone receive an estimated 1.15 and 2.41 million tonnes of plastic waste yearly [1]. Plastics are ubiquitous, durable and often neglectfully discarded, hence plastic waste accumulates and threatens land and marine habitats [2–5]. The impact of plastic waste can be particularly negative in areas of high bio-diversity such as coral reefs; the risk of disease increases from 4% to 89% in corals in close contact with plastics [2]. Plastic waste is also produced in large quantities by research laboratories [6]. In 2014, an estimated 5.5 million tonnes of plastic waste were produced by biological, medical and agricultural research institutions worldwide [6]. Packaging is a by-product of transport and storage and is often insufficiently accounted for and poorly managed [7]. The low rates of recycling and re-usage of packaging leads to its disposal into landfills [7]. The more voluminous a product is, the more packaging it requires.

Cell culture is a flexible tool that can be performed in a variety of dishes and surfaces for diverse types of applications such as drug testing, biomarker identification and expression of recombinant proteins [8–14]. In culture, cells continuously divide and occupy the dish. In order to keep them healthy, cells need to be transferred into new dishes on a regular basis, a process commonly known as cell passage. In this context, single-use plastic consumables are quite convenient and time efficient, however they generate large quantities of biohazardous plastic waste. Here we describe a new cell culture dish designed to reduce the mass and volume of plastic waste generated by cell culture.

## Materials and methods

### Prototype

The outer shell was 3D printed (Ultimaker-2—Ultimaker) with a 2.85 mm, transparent polylactic acid (PLA) filament (1614—Ultimaker). PLA is one of the most commonly used polymers for 3D printing, is biocompatible and is used for biomedical and packaging applications [15]. The layered culture compartments were fabricated with 200 μm food-approved polyethylene terephthalate (PET) sheets (Panduro Hobby AB—672079). These sheets were vacuum formed, rinsed in distilled water, dried, oxygen plasma treated for 2 min at 20% O_2_ (Cute plasma system;—Femto Science), left resting for 4 days in a clean environment, submerged in 70% ethanol for 1 day and finally left to dry for 1 day in a sterile environment. The lid used in the prototype is the lid of a petri dish 100 mm in diameter (430167—Corning Inc.).

### Cell culture

HeLa cells were grown in Dulbecco’s Modified Eagle Medium (DMEM) media (41966–029—Gilbco), 10% fetal bovine serum (FBS) (10270–106—Gilbco) and 1% Penicillin-Streptomycin (15140–122—Gibco). 0.6 million cells were seeded into the first layer of the prototype. After 72 h the cells were trypsinized, washed and a sample stained with trypan blue (T8154—Sigma) 1:1. The cells were analyzed for cell number and percentage of live cells in a TC20 automated cell counter (Bio-Rad Laboratories) according to the manufacturer’s instructions. The first layer, now empty, was removed with the help of a sterile tweezer. From the cells grown in the first layer, 0.6 million cells were seeded into the second layer and the process was repeated. As a control, the same procedures were conducted in three consecutive 100mm in diameter petri dishes (430167—Corning Inc.). In total, six prototypes and six groups of three petri dishes were used to assess cell number and percentage of live.

### Cell staining

Cells were cultured as described above. After 72 h, the media was discarded, the cells quickly rinsed in phosphate-buffered saline (PBS) solution (pH 7.4) and fixed in 4% formaldehyde (1.04003.2500 –MilliporeSigma) in PBS (pH 7.4) for 20 min at RT. Then, the cells were permeabilized in 0.01% triton in PBS (pH 7.4) for 15min at RT, blocked in 3% bovine serum albumin (BSA) in Tris-buffered saline (TBS) solution (pH 7.6) for 1h at 4°C and incubated ON at 4° C with a FITC anti-beta actin antibody (ab6277—Abcam) 1:100 in 3% BSA in TBS (pH 7.6). The cells were washed 3 times for 5 min in PBS (pH 7.4) at RT and incubated for 5 min with Hoechst 33342 (ab228551—Abcam) 1:5000 in PBS (pH 7.4) at RT. Finally, the cells were washed 3 times for 5 min in 0.1% Tween in PBS (pH 7.4) at RT and rinsed 1 time in PBS (pH 7.4) at RT.

### Microscopy

Images of stained cells on the control petri dishes and prototypes were acquired using a Nikon Ti-E Eclipse microscope with a Prior H117 motorized stage, controlled using Micro-Manager[16]. 3 petri dishes and 3 prototype layers were used to assess the number of cells per mm^2^. A set of 15 x and y coordinates were randomly generated and used for each dish. At each random location, fluorescence imaging was carried out using a Nikon Plan Fluor 10x/0.3 objective with a Lumencor SOLA SE II Solid State White Light Engine for illumination along with Semrock LED-DAPI-A-000 and GFP-3035D-000 single-band filter sets. Images were captured and saved using an Andor Zyla 5.5 sCMOS USB3 camera and the number of nuclei present in each image was counted using CellProfiler [17]. The images were prepared for publication with ImageJ. The number of cells per mm^2^ was calculated based on the size of each image (1.011 mm^2^).

### Prototype weight and volume

The weight was assessed in six individual control petri dishes (430167—Corning Inc.) with their respective lids. The weight of each petri dish was multiplied by three, since one prototype has a culture area equivalent to three petri dishes. The weight of six prototypes was also assessed, where each prototype was composed of a lid, three layers and one shell. Since the dishes and prototypes are cylinder-shaped, their external volume was estimated based on radius and height.

### Statistics

One-way ANOVA was used to analyse the results of: cell number, percentage of live cells and number of cells per mm^2^. Unpaired t-test was used to analyse the results of: dish weight and dish volume.

## Results and discussion

**Fig 1:**
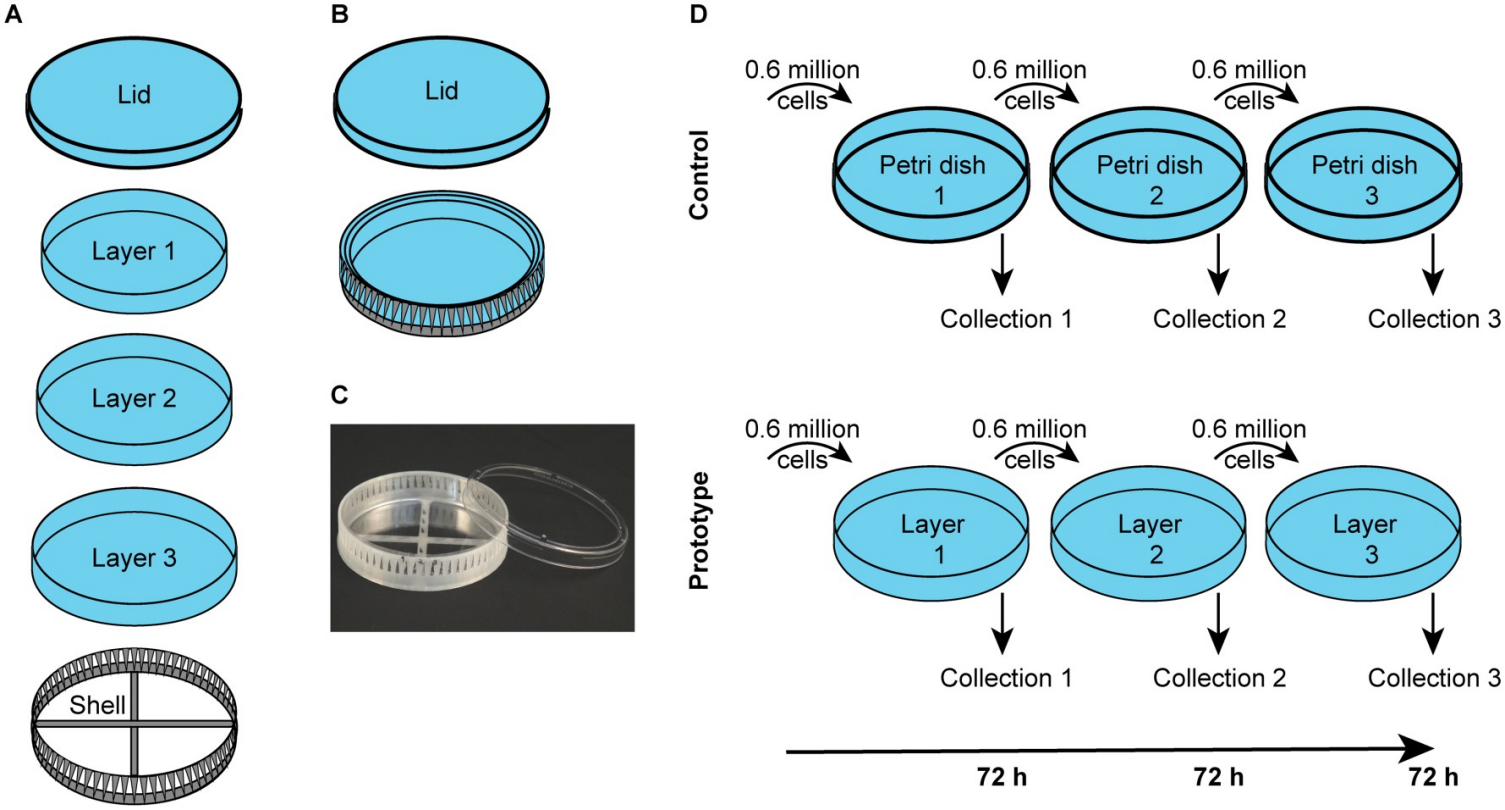
Features of the prototype and experimental layout. (A) The prototype is composed of a lid, three culture layers and a structural outer shell. (B) The layers fit inside each other in order to make the prototype compact. (C) Photo of the prototype. (D) 0.6 million cells were seeded into each dish at the beginning of each expansion. After 72 hours the cells were trypsinized and a sample collected for analysis. The same principle was applied to the prototype using its three layers.

The prototype is composed of a lid, three culture layers and a structural outer shell (Fig 1A). The culture layers are transparent PET containers with a thickness of 200 μm. The outer shell is a net-like PLA structure engineered to provide rigidity, hence facilitating the handling of the prototype as a whole. The lid covers all the layers when in place and allows access to the cells when removed, similarly to a regular petri dish. The layers of the prototype fit inside each other and inside the shell in order to reduce the outer volume of the structure (Fig 1B and 1C). The prototype was tested by seeding 0.6 million HeLa cells into the first layer. After 72 hours the cells were trypsinized, a sample collected and 0.6 million cells were transferred into the second layer. The process was repeated as shown in Fig 1D. As a control, the same procedure was used in groups of three consecutive petri dishes (Fig 1D).

**Fig 2:**
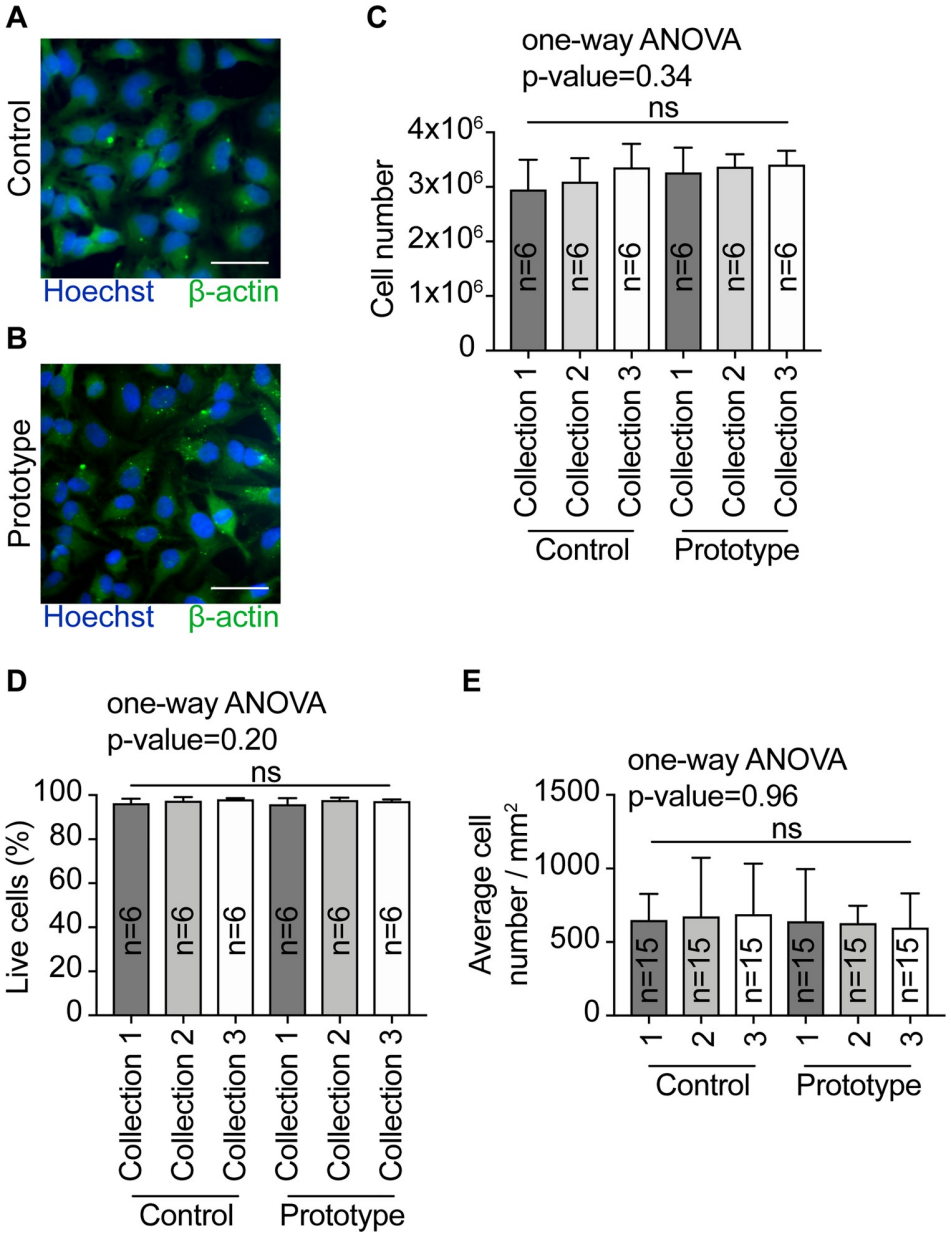
Cell analysis. (A) Cells on the control petri dish after 72 h of culturing. (B) Cells on the prototype after 72 h of culturing. (C) No statistical differences were found between the samples collected from the controls and prototypes in terms of cell number. (D) No statistical differences were found between the samples collected from the controls and prototypes in terms of percentage of live cells. (E) No statistical differences were found between the controls and prototypes in terms of average cell number per mm^2^. Scale bar = 50 μm. Plots: mean and one standard deviation.

Cells presented a similar morphology in both the control dish and the prototype after 72 h of culturing (Fig 2A and 2B). Cells were collected after each expansion to evaluate cell number and percentage of live cells. The different cell collections yield in average 3.2 million cells and we found no statistical differences (one-way ANOVA, p-value = 0.34) between the controls and prototypes in terms of cell number (Fig 2C and S1 Table). On average 97.2% of the cells were alive across the different collections and no statistical differences (one-way ANOVA, p-value = 0.20) were found between the controls and prototypes in terms of percentage of live cells (Fig 2D and S1 Table). Additionally, cells seem to have a similar distribution on the dishes’ surfaces, averaging 651 cells per mm^2^ (Fig 2E and S1 Table). No statistical differences (one-way ANOVA, p-value = 0.96) were found between the controls and the prototypes in terms of average cell number per mm^2^ (Fig 2E and S1 Table). Despite their different designs, petri dishes, cell culture flasks and a variety of multi-well plates are all suitable dishes for cell culture. Altogether, our results indicate that the cells in the prototype performed as well as in a common petri dish, hence validating our design.

**Fig 3:**
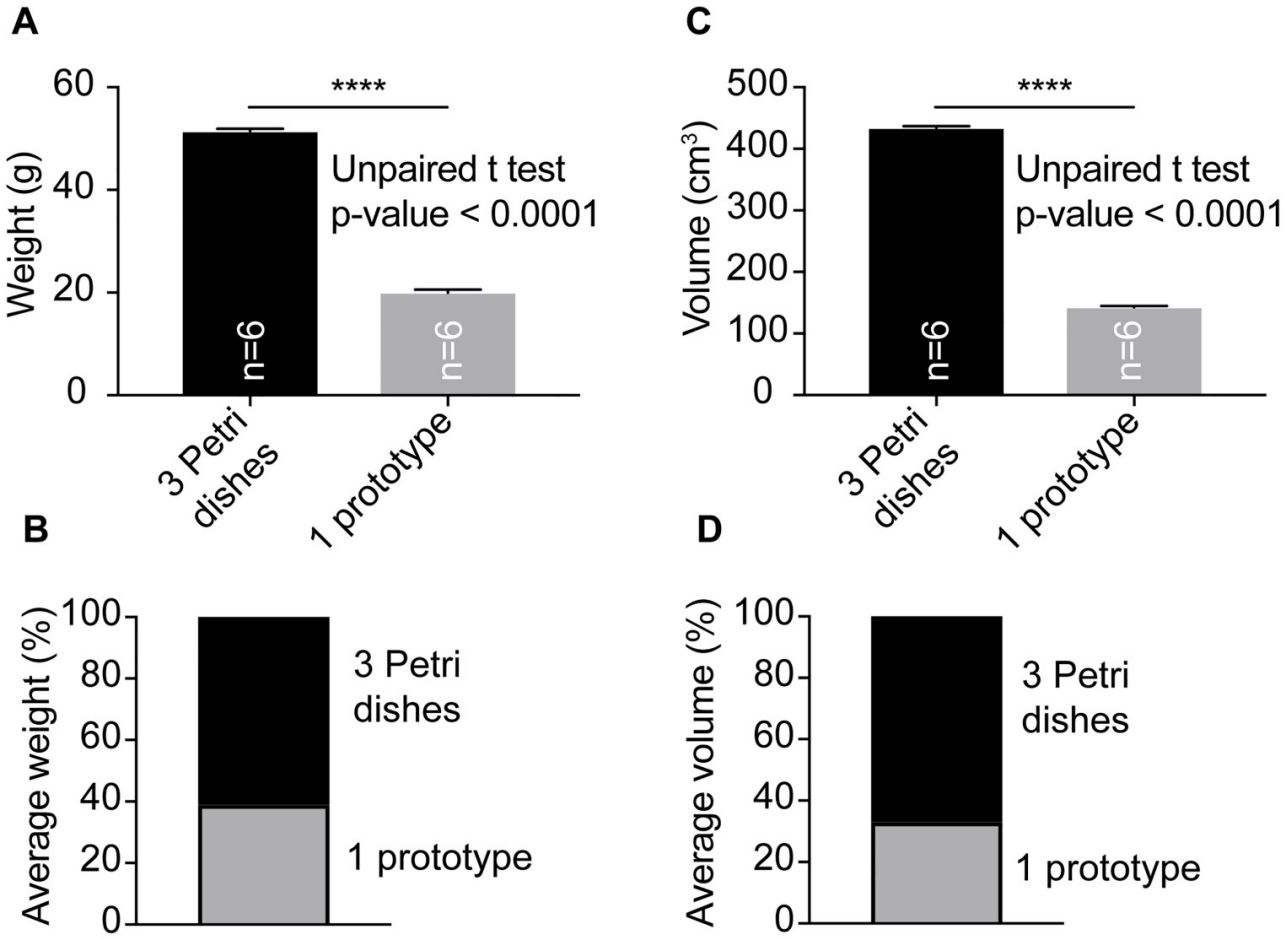
Weight and volume. (A) One prototype has a culture area equivalent to three petri dishes, but it is significantly lighter. (B) The prototype is on average 61% lighter than three petri dishes. (C) One prototype has a culture area equivalent to three petri dishes, but it occupies a significantly smaller volume. (D) The prototype occupies on average 67% less volume than three petri dishes. Plots: mean and one standard deviation.

Current solutions for cell culture are typically single-use sturdy-made (e.g. 1 mm thick) plastic containers. Our prototype, which weighs on average 20.0 grams, has a culture area equivalent to three petri dishes but three petri dishes weigh on average 51.4 grams. Thus, the prototype is 61% lighter than the control (Fig 3A and 3B and S1 Table). The difference in weight between the prototype and control is highly significant (unpaired t-test, p-value < 0.0001) (Fig 3A and 3B and S1 Table). It is likely that the presented dish design could be made using a different material such as polystyrene, a frequently used polymer for cell culture dishes, and in that case a different weight would be expected. Nonetheless, it seems clear that the lightness of our prototype is mainly due to its design: the usage of thin (200 μm) culture layers, a single lid (for all culture layers) and a net-like outer shell.

The prototype is on average 142.8 cm^3^ but, as described above, it has a culture area equivalent to three petri dishes, which have a combined average volume of 434.0 cm^3^. Hence the prototype is on average 67% more compact than the control, a difference that is highly significant (unpaired t-test, p-value < 0.0001) (Fig 3C and 3D and S1 Table). A less voluminous dish should require less packaging material and occupy less space, potentially reducing the carbon emissions associated with its transport and with the disposal of the packages. Additionally, it could reduce the volume of biohazardous plastic waste associated with the disposal of single-use cell culture dishes.

## Supporting information

S1 Table Raw data. (XLSX)
